# First person – Claudia Müller-Sánchez and María Gertrudis Muñiz-Banciella

**DOI:** 10.1242/dmm.052900

**Published:** 2026-04-02

## Abstract

First Person is a series of interviews with the first authors of a selection of papers published in Disease Models & Mechanisms, helping researchers promote themselves alongside their papers. Claudia Müller-Sánchez and María Gertrudis Muñiz-Banciella are co-first authors on ‘ An innovative *in vitro* model for studying the biology of cardiac fibroblasts originating from the epicardium’, published in DMM. Claudia is an Assistant Professor in the lab of Ofelia Martínez-Estrada at the University of Barcelona, Barcelona Spain, investigating the development and application of *in vitro* cellular models to investigate molecular mechanisms in physiological and pathological processes, bridging basic cell biology and translational innovation. María Gertrudis is a PhD student in the same lab, focusing on understanding the cellular and molecular mechanisms that drive cardiac remodelling, and fibrosis in health and disease.

**Figure:**
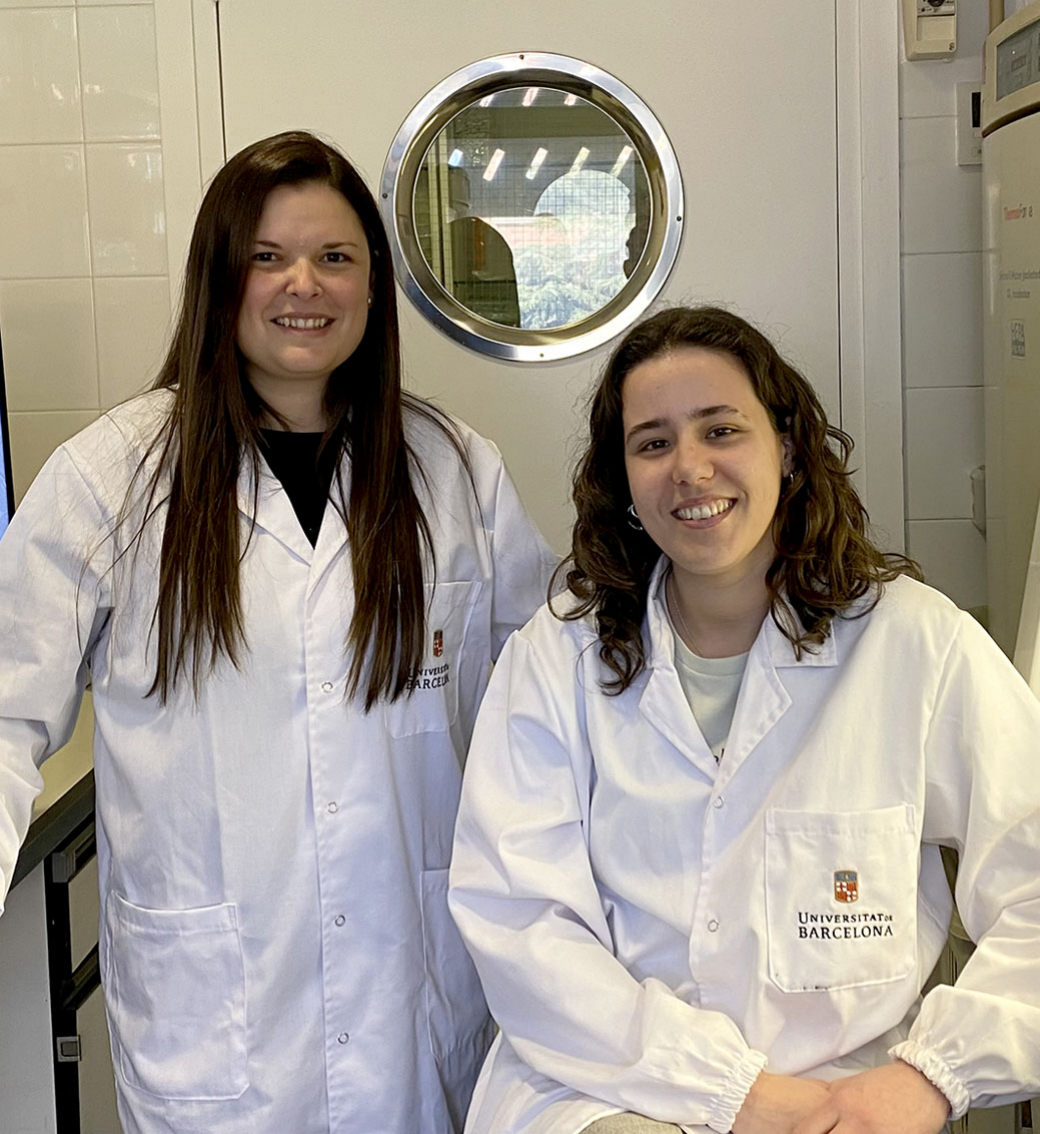
Claudia Müller-Sánchez (left) and María Gertrudis Muñiz-Banciella (right)

**Who or what inspired you to become a scientist?**

**C.M.-S.:** From a very young age, I was fascinated by all forms of life. I lived with my parents in a place surrounded by nature and spent hours observing flowers, seeds, insects and birds, constantly wondering how all of it had come to be. Family dinners with my uncles, some of whom were doctors and scientists, further sparked my curiosity as they discussed various pathologies, treatments and scientific topics. One day, while trying to register for the university entrance exam, I got lost and ended up in a biology lab. A professor leading the practical session asked if I wanted to look at cells under the microscope. Seeing them for the first time, it was magical. In that moment, I knew I had found my calling.

**M.G.M.-B.:** My path into science wasn't driven by a clear childhood calling but by curiosity. I always enjoyed science, especially biology, at school, and became fascinated by how the body works in health and disease. In many ways, I could have ended up in different professions but science gave me a way to turn that curiosity into something meaningful. Being able to ask questions every day and search for answers is what ultimately drew me to this path.

**What is the main question or challenge in disease biology you are addressing in this paper? How did you go about investigating your question or challenge?**

**C.M.-S./M.G.M.-B.:** A major challenge in cardiovascular disease biology is understanding how cardiac fibroblasts drive tissue repair and pathological remodelling after injury. Many injury-induced responses reactivate developmental programs that operate during embryonic heart formation. We, therefore, asked whether we could develop a model to study fibroblasts across both developmental and disease contexts. Most cardiac fibroblasts originate from the embryonic epicardium and play essential roles in heart development and injury responses, yet they remain difficult to study due to the lack of specific markers and their intrinsic heterogeneity. To overcome this limitation, we generated a mouse model that simultaneously labels WT1-derived cells and cells actively expressing WT1, enabling precise lineage tracing of epicardium-derived fibroblasts. By using this approach, we isolated a defined fibroblast population and established a stable immortalized cell line. This *in vitro* platform, which allows deletion of specific genes in a fibroblast-specific manner, complements our *in vivo* models at the cellular and molecular levels. Importantly, it reduces the need for animal experimentation while enabling standardized analyses of fibroblast activation, identity, functional properties, and the mechanisms driving cardiac fibrosis and remodelling. Although our study focuses on the heart, this strategy provides a framework that can be extended to fibroblasts in other organs.

Many injury-induced responses reactivate developmental programs that operate during embryonic heart formation

**How would you explain the main findings of your paper to non-scientific family and friends?**

**C.M.-S./M.G.M.-B.:** When the heart is injured, certain cells called fibroblasts act like ‘repair workers’, trying to fix the damage. We study these cells because, although they are essential for healing, they can sometimes produce too much scar tissue, which can affect how well the heart works. Since it is very hard to observe cardiac fibroblasts directly inside the body, we isolated them and grew them in the lab, giving us a simple and controlled way to focus on this specific cell type. This gives researchers a simpler and more reliable way to study what these cells do, how they behave under injury-related cues and to test potential treatments that could improve recovery after heart disease.

**What are the potential implications of these results for disease biology and the possible impact on patients?**

**C.M.-S./M.G.M.-B.:** By providing a robust and reproducible cardiac fibroblast model, our work offers a useful tool for studying the cellular mechanisms that drive fibrosis and pathological remodelling in the heart. Because excessive scar formation contributes to heart failure and poor recovery after injury, understanding how fibroblasts become activated is a key step towards identifying new therapeutic targets. In the long term, models like ours can help accelerate basic and translational research, enabling faster testing of molecular pathways or potential drugs that modulate fibroblast behaviour and, ultimately, improve the outcomes for patients with cardiovascular disease.

**Figure:**
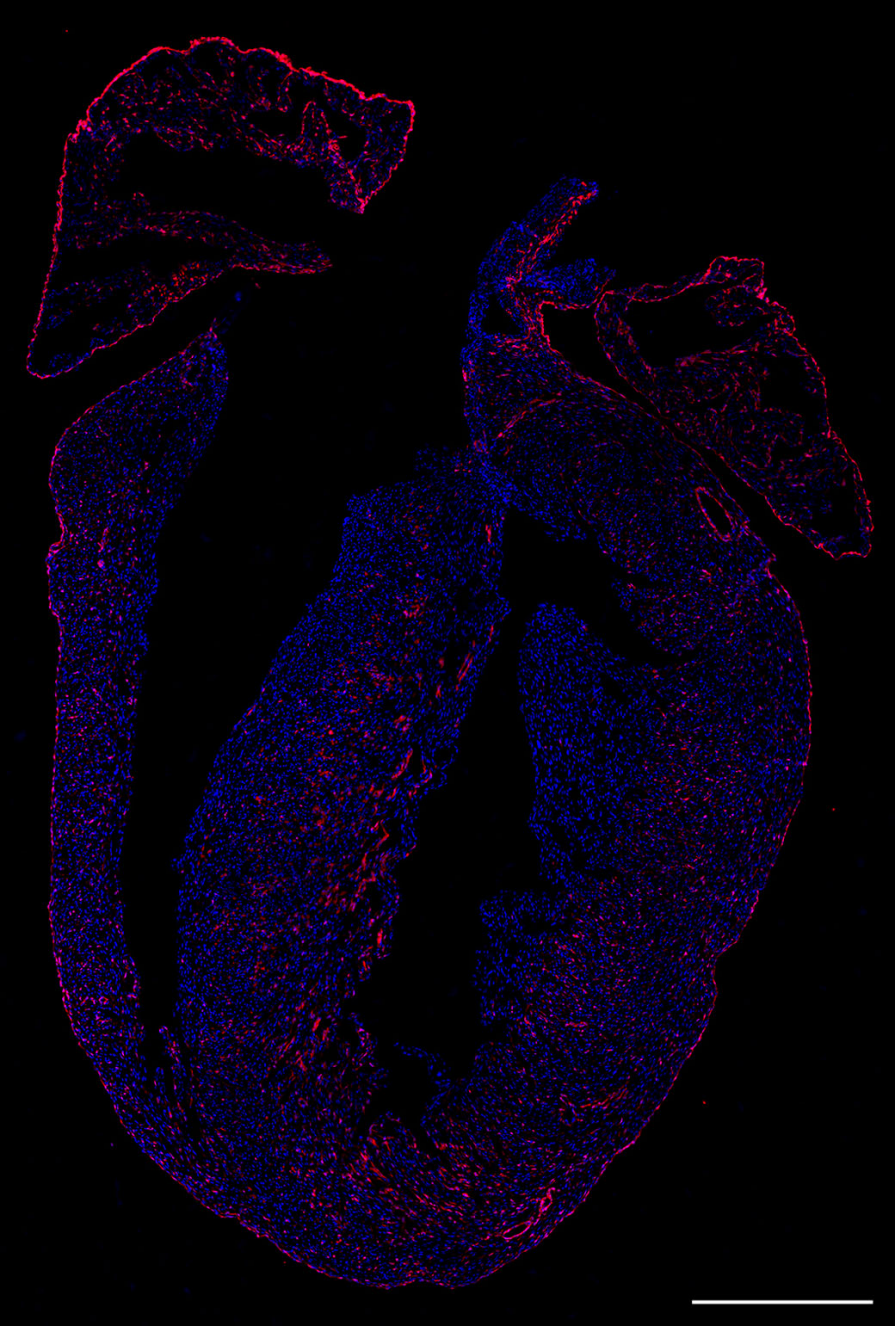
**Wt1Cre efficiently labels WT1 lineage-derived cells in the early postnatal heart.** Immunostaining for red fluorescent protein (RFP, red) on early postnatal heart section from Wt1^GFP/+^;Wt1Cre;ROSA26-tdRFP mice, in which RFP traces WT1-derived cells. Nuclei were stained with DAPI (blue). Scale bar: 500 µm.

**Why did you choose DMM for your paper?**

**C.M.-S./M.G.M.-B.:** We chose Disease Models & Mechanisms for its strong focus on biologically relevant models that bridge basic research and disease mechanisms. The journal was initially suggested by Dr Ofelia Martínez-Estrada, and we collectively agreed it would be the best fit for our work, since it provides a robust cellular model to study cardiac fibroblast behaviour and fibrosis, aligning well with the journal's translational focus. At the University of Barcelona, researchers are encouraged to publish in Open Access journals, and we appreciated that DMM is published by The Company of Biologists, a non-profit organization committed to supporting and inspiring the global biological research community. Its focus on technically solid, broad and useful resources made DMM a natural and ideal home for our study, perfectly aligning with our goal to offer a practical resource for the wider research community.

At the University of Barcelona, researchers are encouraged to publish in Open Access journals, and we appreciated that DMM is published by The Company of Biologists, a non-profit organization committed to supporting and inspiring the global biological research community

**Given your current role, what challenges do you face and what changes could improve the professional lives of other scientists in this role?**

**C.M.-S.:** In my current role as an Assistant Professor at the University of Barcelona, one of the main challenges is balancing research, teaching, student supervision and administrative duties. Managing this combination of tasks can limit the time available to advance scientific work. Clearer career-progression pathways, protected research time and access to structured mentorship and training would significantly support early-career researchers. Streamlined administrative processes and more stable funding schemes would also improve productivity and overall working conditions.

**M.G.M.-B.:** As a PhD student, securing fellowships or contracts is often highly competitive and uncertain, which makes it difficult to plan both research projects and personal life. Increasing the number of funded positions and offering more-stable career paths for young scientists would improve this stage of our careers and, definitely, would make academia more sustainable and inclusive for the next generation of researchers.

**What's next for you?**

**C.M.-S.:** Looking ahead, my priority is to consolidate my scientific leadership within the ‘*in vitro* modelling’ research line at Celltec-UB and to promote its growth, visibility and impact. I aim to further expand the development and application of *in vitro* cellular models, to investigate key cellular and molecular mechanisms in both physiological and pathological contexts. A central objective will be to enhance the translational potential of these models by fostering new collaborations and developing tools with applicability across the biomedical, agri-food, cosmetic and pharmaceutical sectors. Securing long-term funding and establishing a robust, competitive research programme will be essential to achieving these goals. Ultimately, my aim is to build a sustainable and impactful research line that contributes meaningfully to fundamental cell biology and to applied biomedical innovation.

**M.G.M.-B.:** For my next steps, I will continue working on my PhD research, moving from *in vitro* studies to *in vivo* approaches, where we aim to characterize our knockout mouse model in more detail. In particular, we are interested in understanding how cardiac fibroblasts behave when WT1 function is modulated, and how this affects cardiac remodelling and fibrosis. Combining cellular models with *in vivo* analyses will help us build a more complete picture of fibroblast function in heart development and disease.

**Tell us something interesting about yourself that wouldn't be on your CV**

**C.M.-S.:** Perhaps the most curious thing about me is my passion for children's literature. I am fascinated by how these books can explain complex topics in a simple and creative way, without putting any limits on imagination. They always reflect this childlike curiosity that connects me with my inner child while, at the same time, I appreciate their educational role, teaching young readers to learn and question the world from an early age.

**M.G.M.-B.:** Outside the lab, I love live music and going to concerts whenever I can. I'm especially into Spanish rock and punk, and some of my favourite weekends are spent travelling to small festivals in remote towns across Spain. I recently started learning to play the drums, which has been a fun and completely different way to challenge myself and switch off from the lab.
